# Determinants of wealth-related inequalities in full vaccination coverage among children in Nepal: a decomposition analysis of nationally representative household survey data

**DOI:** 10.1186/s12889-024-19456-z

**Published:** 2024-07-25

**Authors:** Barun Kumar Singh, Resham B. Khatri

**Affiliations:** 1 Health Nutrition Education and Agriculture Research Development, Saptari, Nepal; 2 School of Public Health, University of Queensland, Brisbane, Australia

**Keywords:** Full vaccination, Children, Inequality, Decomposition analysis

## Abstract

**Background:**

Over the past two decades, child health indicators in Nepal have improved significantly at the national level. Yet, this progress hasn’t been uniform across various population subsets. This study identified the determinants associated with childhood full vaccination, assessed wealth-related inequalities, and delved into the key factors driving this inequality.

**Methods:**

Data for this study were taken from the most recent nationally representative Nepal Demographic and Health Survey 2022. A total of 959 children aged 12–23 months who had received routine childhood basic antigens as per the national immunisation program were considered for analysis. Binary logistic regression models were conducted to identify the associated factors with outcome variable (uptake of full vaccination). The concentration curve and Erreygers normalized concentration index were used to assess inequality in full vaccination. Household wealth quintile index scores were used to measure wealth-related inequality and decomposition analysis was conducted to identify determinants explaining wealth-related inequality in the uptake of childhood vaccination.

**Results:**

The coverage of full vaccination among children was 79.8% at national level. Several factors, including maternal health service utilisation variables (e.g., antenatal care, institutional delivery), financial challenges related to visiting health facilities, and mothers’ awareness of health mother group meetings within their ward, were associated with the uptake of full vaccination coverage among children. The concentration curve was below the line of equality, and the relative Erreygers normalized concentration index was 0.090, indicating that full vaccination was disproportionately higher among children from wealthy groups. The decomposition analysis identified institutional delivery (20.21%), the money needed to visit health facilities (14.25%), maternal education (16.79%), maternal age (8.53%), and caste (3.03%) were important contributors to wealth related inequalities in childhood full vaccination uptake.

**Conclusions:**

There was notable wealth-related inequality in full vaccine uptake among children in Nepal. Multisectoral actions involving responsible stakeholders are pivotal in reducing the inequalities, including promoting access to maternal health services and improving educational attainment among mothers from socioeconomically disadvantaged communities.

**Supplementary Information:**

The online version contains supplementary material available at 10.1186/s12889-024-19456-z.

## Background

Child health improvement has been a global public health agenda for the last four decades [1]. Reducing child mortality has been a priority of global policy agendas including Sustainable Development Goals (SDGs) [2]. Immunisation is often regarded as a cost-effective public health intervention that can avert an estimated 4–5 million deaths in all age groups yearly from vaccine-preventable diseases (VPD) worldwide [3]. Moreover, 1.5 million deaths could be avoided if global vaccination achieved universal coverage [3]. The Global Vaccine Action Plan (GVAP) targeted 95% national coverage of the third dose of diphtheria, pertussis, and tetanus (DPT 3), vaccine a globally recognized proxy for vaccination system performance, and at least 80% DPT 3 coverage for subnational levels by 2015 [4]. Despite ongoing efforts, there was only an estimated 84% global DPT 3 coverage in 2022, leaving an estimated 20.5 million children incompletely vaccinated. Furthermore, the number of children who received no vaccinations has, concerningly, been trending upward, from 12.9 million in 2019 to 14.3 million in 2022, causing a decline in full vaccination coverage [5].

Historically, vaccination coverage has been measured by the proportion of children receiving all “basic” antigens. “A child is considered fully vaccinated against all basic antigens if they have received the Bacille Calmette-Guerin (BCG) vaccine, three doses of oral polio and DPT-containing vaccine, and a single dose of the measles-rubella (MR) vaccine” [6]. The proportion of children receiving all basic antigens is considered an important measure of vaccination coverage. In Nepal, several health policies, programs, strategies, and services related to immunisation programs are designed and implemented in Nepal aligning with global commitments. The BCG vaccine is given at birth or first clinic contact, while the OPV and DPT (given as pentavalent: DPT-HepB-Hib) vaccines are given together at 6, 10, and 14 weeks of age. The first dose of the MR vaccine is given at or soon after nine months, whereas the second dose is given at 15 months of age [6–8]. Nepal’s National Immunization Program (NIP) has made huge progress and is often regarded as one of the most successful public health programs [9]. Since its inception, this program has made significant achievements in controlling, eliminating, and eradicating several VPDs. For example, some successful examples of immunisation programs in Nepal include the eradication of smallpox (1977), elimination of maternal and neonatal tetanus (MNT) (2005), polio-free certification (2014), rubella control certification (2018), and certification of Hepatitis B control in children through immunisation (2019) [10]. Access to vaccination has improved in hard-to-reach areas and marginalised populations, including children from the lowest wealth quintile [11]. Furthermore, Nepal introduced the unique full immunisation declaration program in 2012/2013, with support from external development partners, and accelerated coverage and access to routine childhood vaccines by implementing full immunisation declaration guidelines [12]. As of November 2023, 72 out of 77 districts and 724 out of 753 local levels have achieved ‘full immunisation’ status [10].

These policy and programmatic efforts on childhood vaccination have contributed to the ongoing decline in child mortality, as evidenced by periodic survey reports. For instance, Nepal substantially improved under-five, infant, and neonatal mortality over the last two and half decades. Under-5, infant, and neonatal mortality declined by 72%, 64%, and 58%, respectively, between 1996 and 2022. Recent NDHS 2022 data indicate that the under-5 mortality rate (U5MR), infant mortality rate, and neonatal mortality rate are 33, 28, and 21 deaths per 1000 live births, respectively [6]. However, there is a significant socioeconomic disparity in the under-5 mortality rate, with 16 deaths per 1000 live births in the highest wealth quintile compared to 53 deaths per 1000 live births in the poorest wealth quintile [6]. The NIP has been crucial in preventing several VPDs responsible for avoidable under-five deaths [6, 8]. However, specific population groups in Nepal still have low coverage of full vaccination, with recent surveys indicating a decrease in full vaccination coverage [6]. Some marginalised groups, including internal migrants, neighbouring nearby India, specific ethnicities, low socioeconomic status, urban poor, and slum populations, face limited access to routine childhood vaccines [8]. These disparities in the utilization of immunisation services may present additional hurdles to universal coverage of vaccine uptake. Determinants of childhood vaccination coverage in low- and middle-income countries (LMICs), including Nepal, are not in limited numbers and are usually of a complex nature [13]. Household characteristics, including maternal education, socioeconomic status, and caste/ethnicity, are important predictors.

Moreover, factors associated with the immunisation service in Nepal, such as physical access to health facilities, availability of health professionals, cold chain maintenance system, and direct and indirect costs associated with vaccinations, also substantially impact vaccine uptake [9, 14, 15]. Most studies in LMICs, including Nepal, have documented inequality in vaccination coverage, favouring the privileged groups such as households with higher wealth status or maternal education [11, 16–21]. Children from poor households are more likely to experience a decline in vaccination coverage, making them more susceptible to VPDs and their consequences. This vulnerability can lead to catastrophic health expenditures, driving the affected households further into poverty [22, 23]. It is pivotal to examine vaccination inequality to achieve a relatively fair distribution of health outcomes. Appropriate strategies are needed to improve childhood vaccination coverage among children from disadvantaged communities and hard-to-reach areas. This aligns with the WHO’s goal of making immunization services accessible to everyone (leaving no one behind) [24] and the target of SDG3 of reducing U5MR by 2030 [25].

Assessing inequalities in full vaccination coverage, identifying gaps in routinely delivered immunisation services, and gathering valuable information to roll out effective strategies and policies are essential. Tracking those children who did not receive full vaccinations is important to develop an equity-oriented immunisation program to reach disadvantaged populations and reduce Nepal’s vaccine-preventable childhood morbidities and mortalities. Although previous studies in Nepal have addressed factors associated with full vaccination [11, 19, 26, 27], and another study [21] assessed the inequality of vaccination. Nonetheless, limited evidence is available on decomposition analysis of the determinants of socioeconomic inequality based on nationally representative data. The objective of this paper is threefold: (i) to analyse the determinants of full vaccination among children in Nepal using the data from the most recent nationally representative household survey; (ii) to measure socioeconomic inequality in the uptake of full vaccination; (iii) to identify the main components that explain socio-economic inequality in full vaccination uptake. Findings of this study provide important insights for the policymakers to develop evidence-based policy measures to address the inequitable distribution of these immunisation related cost-effective life-saving interventions.

## Methods

### Study population and data source

This study used the cross-sectional data from the NDHS 2022 survey from January 5th to June 22nd, 2022. The survey collected nationally representative samples from all seven provinces, further stratified by urban and rural areas. This survey used an updated urban and rural classification system based on structural changes made in the country on 17 April 2017 (post-federalization), which changed the nation into a pro-urban, accommodating 65% of the population. The NDHS adopted two stage sampling approach. In the first stage, 476 primary sampling units (PSUs) were selected using probability proportional to size, with 248 PSUs from urban areas and 228 from rural areas. In the second stage, 30 households were selected from each PSU, resulting in a total sample size of 14,280 households, consisting of 7,440 urban and 6,840 rural households. From these sampled households, 15,238 women aged 15–49 were eligible for interviews. Of these, 14,845 women were interviewed, yielding a response rate of 97.42%. The sample included 5,205 children under five years whose mothers were interviewed to determine vaccination status. Detailed sampling approach is described in original report of the NDHS 2022 [6].

**Fig. 1:**
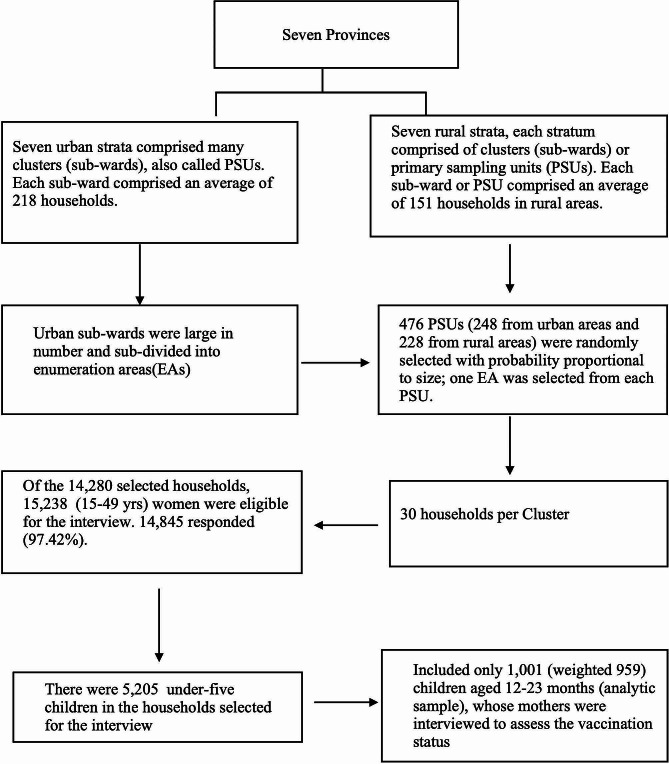
Schematic representation of sampling procedure for study population in NDHS 2022

The source population for this study was all children aged 12–23 months living in Nepal. This study’s term “children” refers to 12–23 months age group. The final sample included in the analysis were 959 children who were alive and living with the mother because they were the youngest children who had reached the age by which they should be fully vaccinated with the basic antigens (Fig. 1). For this study, we extracted the outcome and explanatory variables from the children, women, and household datasets of NDHS 2022. The survey data was collected using a structured and pretested questionnaire. Details about the sampled mother and children’s pair were collected using a woman’s questionnaire that primarily focused on the respondents’ background characteristics, vaccination and childhood illness, reproduction, and contraception. Another questionnaire was used to collect the sociodemographic details of everyone in the family, including inventory and asset information. Comprehensive information about the data collection tools and techniques for NDHS 2022 is available elsewhere [6].

### Study variables

The outcome variable of this study was children’s vaccination status with binary categories: “Fully Vaccinated” and “Not Fully Vaccinated.” The study considered a child who received one dose of the vaccine against tuberculosis (BCG), three doses each of the pentavalent (DPT-Hib, Hep-B) and OPVs, and a first dose of the Measles-Rubella (MR) vaccine any time before data collection as “fully vaccinated” and coded as “Yes = 1.” Conversely, a child who missed at least one dose of the recommended basic vaccines or didn’t receive any vaccines was considered “not fully vaccinated” and coded as “No = 0”. Before creating the outcome variable with two categories, We recoded the responses for each of the eight vaccine doses to indicate whether each dose was “vaccinated” or “not vaccinated” and then combined these to reflect “fully vaccinated.” The study obtained the vaccination status of children from the written vaccine records or the mother’s verbal report if cards were unavailable.

**Fig. 2:**
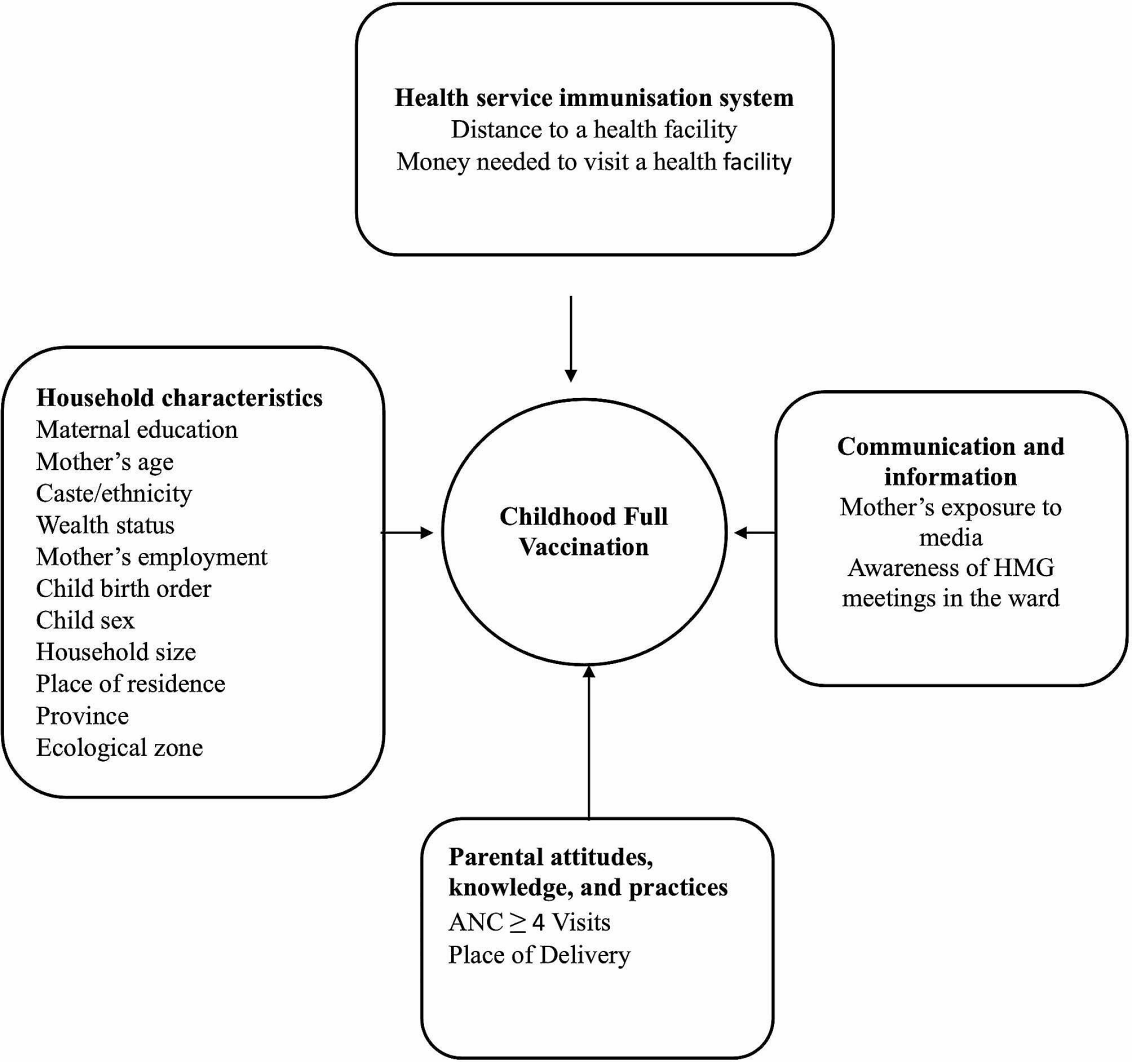
Conceptual framework adapted from the WHO framework on the epidemiology of nonvaccinated child

All seventeen explanatory variables of interest were based on the literature review [11, 15, 18, 21, 26–34] and the variables available in the dataset. The WHO framework on the epidemiology of nonvaccinated children [35–37] describes the factors influencing childhood vaccination into four main categories (Fig. 2): (a) health service immunisation system; (b) communication and information; (c) household characteristics; and (d) parental attitude, knowledge, and practices. In our study, the immunisation system category comprised the distance to a health facility and the money needed to visit a health facility. The communication and information category comprised exposure to mass media and awareness of HMG in the ward. Household characteristics included the following variables: maternal education, mother’s age, caste/ethnicity, wealth status, mother’s employment (in the past 12 months), birth order, child sex, household size, place of residence, province, and ecological zone. Variables such as ANC ≥ 4 visits and place of delivery represented the parental attitudes, knowledge, and practices.

Details about the description of categories of explanatory variables are explained in Supplementary Table S1. Socioeconomic status was measured using a household wealth index, a proxy measure of inequality without income, expenditure, and consumption data [38]. According to NDHS 2022, the household wealth index was derived using principal component analysis. Scores were assigned based on easily collectible data on household consumer goods (such as televisions, bicycles, and cars) and household characteristics (housing construction materials, water access, and sanitation facilities). Household wealth quintiles were then computed by assigning a household score to each household member, ranking each person in the household population by their score, and dividing the distribution into five equal categories comprising 20% of the population [39]. As the wealth index offers an ordinal interpretation, it served as a household ranking variable for measuring the inequality of childhood full vaccination [6].

### Statistical analyses

The characteristics of the mother-child pairs included in this study were described using frequency and percentage. We followed Hosmer and Lemeshow’s (2000) [40] incremental process for specifying the initial model, refining the set of predictors, and determining the final form of the logistic regression model [41]. Multivariable binary logistic regression analysis examined the associations of full vaccination status (Yes/No) simultaneously with other variables. The study expressed the results as an odds ratio with a 95% confidence interval (CI). We ran the model fitness test using multiparameter Wald tests to determine the overall significance of each predictor. The multivariable binary logistic regression model included all the explanatory variables in the conceptual framework (Fig. 2), which we considered important based on previous studies [11, 15, 18, 21, 26–34]. The study dropped variables such as ecological zones following the multicollinearity check with a variance inflation factor (VIF) ≥ 5 [42]. Details about the VIF are presented in the supplementary Table 2. The Pearson goodness of fit test for the model accounting for survey design gave *p* = 0.873 (F-adjusted test statistic:0.50) with all the covariates in the multivariable model, which indicated no evidence of poor fit. Since the NDHS survey employed a two-stage stratified cluster sampling technique, the recommended sample weights provided by the NDHS 2022 were used for the analysis [6]. We adjusted the analysis by incorporating the sample weights, primary sampling units, and strata using the “SVY” command in STATA 18 to account for the complex survey design, which underestimated the variance.

### Inequality measurement

The concentration curve (CC) and concentration index (CIX) in their relative formulation were employed to examine the inequality in the use of health services (childhood vaccination) across the socio-economic characteristics of the mother-child pair [43]. CIX, in its relative formulation, reflects horizontal inequity, assuming equal vaccination needs for all children. The CC plots the cumulative proportion of mothers ranked by wealth index score (x-axis) against the cumulative proportion of fully vaccinated children (y-axis). The 45-degree inclination from the origin showed perfect equality. If the CC coincides with the line of equality, vaccination uptake is equal among children. However, if the CC deviates from the line of equality, inequality in vaccination uptake exists and is biased towards mother-child pairs belonging to either low or high socioeconomic status. Although the concentration curve (CC) offers a graphical representation of inequality, it does not quantify the magnitude of the inequality numerically. Therefore, the Concentration Index (CIX) was utilized in this study to compute the degree of socioeconomic inequality in full vaccine uptake among children aged 12–23 months [21, 22, 44, 45]. The CIX quantifies the magnitude of wealth-related inequality, which is twice the area between the line of equality and CC [43]. The CIX ranges from − 1 to + 1, with zero indicating no inequality. A positive CIX implies a concentration of fully vaccinated children among higher socio-economic groups (pro-rich). In comparison, a negative CIX suggests a concentration among lower socio-economic groups (pro-poor). Provided that the outcome variable (full vaccination-yes/no) is binary, Erreygers’ normalized concentration index (ECIX) recommended for such cases was used in the study to assess the inequality of full vaccination among the children [46–51], as shown in Eq. 1.

1 $$\:ECI=\frac{8*cov\:\left({y}_{i,}{r}_{i}\right)}{b-a}$$

Where $\:{y}_{i}$ is full vaccination uptake, $\:{r}_{i}$ is the socioeconomic status ranking of individual mother-child pairs $\:i$ by wealth index, $\:cov$ is covariance, and ‘b’ and ‘denotes the upper and lower bound of the outcome variable, respectively. The range ($\:b-a)$ become one for binary variables, like in our study. The *glcurve* (Lorenz as option) and *conindex* STATA commands were used to produce the CC and measure the ECIX, respectively [43].

### Decomposition of the concentration index

While the ECI presents the extent of socioeconomic inequality in full vaccination uptake, it does not explain the factors behind these disparities. Identifying these factors is pivotal for formulating effective policy measures. Therefore, we used the decomposition analysis of ECI to identify the explanatory factors contributing to inequality in childhood full vaccinations [45, 52]. The selection of variables for the decomposition of ECIX was based on the results of multivariable binary logistic regression (statistical significance), policy relevance, and literature review of empirical studies [11, 15, 18, 21, 27, 29, 32, 34, 53, 54].

Let’s suppose that our outcome variable of interest, full vaccinee uptake $\:{y}_{i}$, can be stated as a linear function of the explanatory variables as per the following multivariable linear regression Eq. 2.

2 $$\:{y}_{i=}\alpha\:+\:{{\Sigma\:}}_{k}{\beta\:}_{k}{\mathcal{X}}_{ki}+{\epsilon\:}_{i}$$

Where

$\:{y}_{i}$ is full vaccine uptake ($\:{y}_{i}$ = 1 if the child received all recommended eight basic antigens and $\:{y}_{i}=0\:$ otherwise)

$\:{\mathcal{X}}_{ki}$: a set of explanatory variables in the model for full vaccine uptake;

$\:{\beta\:}_{k}$ : regression coefficient of explanatory variables $\:{\mathcal{X}}_{k}$

$$\:{\epsilon\:}_{i}$$ : error term

After fitting the model, the ECIX for full vaccine uptake can be decomposed into the contribution of individual explanatory variables using the Eq. (3) [52].

3 $$\text{ECIX}=\:\left(\genfrac{}{}{0pt}{}{\sum\:}{k}{\beta\:}_{k}{\mathcal{X}}_{k}{C}_{k}+\:G{C}_{\epsilon\:}\right)$$

Where $\:{\beta\:}_{k}$ is a partial regression coefficient of explanatory variables estimated from linear regression provided in Eq. (3), $\:{\mathcal{X}}_{k}$ is the mean of the explanatory variable, and $\:{C}_{k}$ is the concentration index of explanatory variables, and $\:G{C}_{\epsilon\:}$ is generalised concentration index for the error term ($\:\epsilon\:$).

As shown in Eq. 3, ECIX combines the deterministic and residual components. The deterministic component $\:\genfrac{}{}{0pt}{}{\sum\:}{k}{\beta\:}_{k}{\mathcal{X}}_{k}{C}_{k}$ comprises the sum of the contribution of each explanatory variable to inequality in childhood full vaccination uptake. The extent of contribution provided by explanatory variable ($\:{\mathcal{X}}_{ki})$ to inequality varies according to its distribution by socioeconomic status (derived from its concentration index) and how it is associated with full vaccine uptake (measured by its regression coefficient $\:{\beta\:}_{k}$). The greater the value of $\:{C}_{k}$ or $\:{\beta\:}_{k}$ of the explanatory variable, the more significant its contribution to the observed overall inequality. The percentage contribution of each explanatory variable to overall inequality was derived by dividing its contribution by ECIX and multiplying by the hundreds. As Eq. (3) mentioned, the residual component $\:G{C}_{\epsilon\:}$ represents the inequality of full vaccination not explained by a set of explanatory variables in the model. Although binary variables are best estimated by non-linear models, a generalized linear model (identity link) was fitted to our data in Eq. (3) as a linear assumption of decomposition analysis is fulfilled, and the results are easier to interpret relatively with a linear model. To check robustness, we performed the decomposition analysis using partial effects of logistic regression and found fairly consistent results, and the pattern remains unchanged (details are provided in Supplementary Table 4). This was concurrent with the previously published studies [51, 55].

**Table 1:** Background characteristics of mother and child (12–23 months) pair in NDHS 2022

Variables	Frequency (%)^a^	Fully Vaccinated (%) ^b^	*P* Value
**Household characteristics**			
**Province**			< 0.001
Koshi	168(17.5)	80.8	
Madhesh	269(28.0)	67.0	
Bagmati	134(14.0)	83.4	
Gandaki	51(5.3)	93.4	
Lumbini	172(17.9)	85.3	
Karnali	79(8.2)	84.3	
Sudurpaschim	87(9.1)	88.8	
**Place of residence**			0.786
Urban	623(65.0)	79.5	
Rural	336(35.0)	80.3	
**Ecological zone**			< 0.010
Mountain	68(7.1)	89.1	
Hill	301(31.3)	84.3	
Terai	591(61.6)	76.4	
**Sex of child**			0.214
Male	486(50.7)	81.5	
Female	473(49.3)	78.0	
**Maternal education**			<0.001
No Education	197(20.5)	64.8	
Primary	338(35.3)	80.5	
Some Secondary	261(27.2)	85.9	
SLC and Above	163(17.0)	86.4	
**Mother’s age (years)**			0.144
15–19	76(7.9)	72.4	
20–24	399(41.6)	79.1	
25–29	299(31.2)	80.3	
30–34	128(13.3)	87.4	
35–49	57(5.9)	74.3	
**Caste/ethnicity**			<0.001
Brahmin Hill	57(5.9)	90.8	
Chhetri Hill	176(18.4)	86.3	
Terai Caste	187(19.5)	73.3	
Dalit	194(20.2)	69.2	
Hill Janajati	192(20.0)	86.9	
Terai Janajati	87(9.1)	90.8	
Muslim	66(6.9)	67.0	
**Wealth status**			<0.050
Poorest	233(24.3)	75.8	
Poorer	224(23.4)	73.2	
Middle	180(18.8)	85.0	
Richer	193(20.1)	85.2	
Richest	129(13.5)	82.8	
**Household size**			<0.050
Small (one-three)	116(12.1)	90.7	
Medium (four-five)	370(38.6)	80	
Large (≥ Six)	473(49.3)	76.9	
**Birth order**			0.001
One	401(41.8)	81.8	
Two-Three	463(48.3)	81.3	
≥ Four	95(9.9)	64.0	
**Mother’s employment (past 12 months)**			<0.010
No	369(38.5)	73.8	
Yes	590(61.5)	83.5	
**Communication and information**			
**Exposure to mass media**			<0.010
No Exposure	250(26.1)	72.0	
Less than Once a Week	287(29.9)	80.6	
At least Once a Week	422(44.0)	83.8	
**Aware of HMG in ward**			<0.001
No	669(70.0)	76.6	
Yes	291(30.0)	87.2	
**Parental attitude**, **knowledge**, **and practices**			
**ANC ≥ 4 visits**			<0.001
No	231(24.1)	67.9	
Yes	728(75.9)	83.5	
**Place of delivery**			<0.001
Elsewhere*	214(22.3)	69.5	
Health Facility	745(77.7)	82.7	
**Health service immunisation system**			
**Distance to health facility**			<0.010
Big Problem	385(40.1)	75.0	
Not Big Problem	574(59.9)	83.0	
**Money needed to visit health facility**			
Big Problem	389(40.6)	73.2	< 0.001
Not Big Problem	570(59.4)	84.3	
**Total**	**959 (100)**	**79.8**	

Notes: ^a^ percentage reported within the column; ^b^ percentage reported within the row

*elsewhere (respondent’s home, other’s home, and other recoded as elsewhere)

During decomposition analysis, Erreygers and Kessels (2013) argued against including wealth status as an explanatory variable in the multivariable regression model [56]. Using the wealth status makes the residual component approximately null and causes the wealth quintile to dominate the explanation of inequality in full vaccination. Hence, we decided not to use the wealth status as an explanatory variable in the regression model in the decomposition analysis.

## Results

Of the total children (weighted *N* = 959), 79.8% were fully vaccinated, and 20.2% were not. Table 1 presents descriptive statistics for the variables from the health service immunisation system, communication and information, household characteristics, and parental attitude, knowledge and practices, disaggregated by vaccination status. Most of the mothers belonged to Madhesh province, urban areas, terai ecological zone, who had primary education, who were aged 20–24 years, employed in the past 12 months, exposed to mass media weekly, had at least 4 ANC visits, and who delivered in health facilities. Similarly, most perceived financial and distance barriers to accessing health facilities and were unaware of HMG meetings in their ward. Further, most children were born in large households and were in the birth order of two to three. The proportion of fully vaccinated children was highest in Gandaki province, followed by Sudurpaschim, Lumbini, Karnali, Bagmati, Koshi, and Madhesh. Children from mountain ecological zones, wealthier households, and with one to three birth orders were more likely to be fully vaccinated. Similarly, children with mothers exposed to mass media weekly, receiving four or more ANC visits, employed in the past year, and delivering the child in health facilities were more likely to be fully vaccinated. Likewise, children whose mothers didn’t perceive financial or distance barriers to accessing health facilities and were aware of HMG meetings in the ward were also more likely to be fully vaccinated. Excluding place of residence, child sex, and mother’s age, most of the variables in the study showed evidence of association with full vaccination among children.

**Table 2:** Decomposition of concentration index (CIX) of full vaccination in Nepal, NDHS 2022

Variables	Elasticity	Concentration indices (determinants)	Absolute contribution to CIX	Percentage contributions
**Household characteristics**				
**Maternal education**				
No Education	Base	Base	Base	Base
Primary	0.025935	-0.08309	-0.00862	-9.49082
Some Secondary	0.026071	0.07722	0.00805	8.86732
SLC and Higher	0.009536	0.41443	0.01581	17.4062
**Overall**	**0.061541**	**0.40857**	**0.01524**	**16.7827**
**Caste/Ethnicity**				
Brahmin Hill	Base	Base	Base	Base
Chhetri Hill	-0.011831	-0.081438	0.003854	4.243869
Terai Caste	-0.025158	0.159445	-0.016045	-17.667752
Dalit	-0.024453	-0.197573	0.019325	21.279546
Hill Janajati	-0.00374	-0.111964	0.001675	1.844219
Terai Janajati	0.000228	0.130683	0.000119	0.1313
Muslim	-0.008129	0.189701	-0.006169	-6.792526
**Overall**	**-0.073084**	**0.088854**	**0.00276**	**3.038656**
**Mother’s employment (in past 12 months)**				
No	Base	Base	Base	Base
**Yes**	**0.035404**	**-0.123443**	**-0.017481**	**-19.249235**
**Mother’s Age (years)**				
15–19	Base	Base	Base	Base
20–24	0.011092	-0.034949	-0.001551	-1.707357
25–29	0.014926	0.045991	0.002746	3.023511
30–34	0.013916	0.117211	0.006525	7.184443
35–49	0.003176	0.002167	0.000028	0.030312
**Overall**	**0.04311**	**0.13042**	**0.007747**	**8.530909**
**Communication and information**				
**Aware of HMG meeting in ward**				
No	Base	Base	Base	Base
**Yes**	**0.020701**	**-0.142111**	**-0.011767**	**-12.957132**
**Parental attitude**, **knowledge**, **and practices**				
**ANC ≥ 4 Visits**				
No				
**Yes**	**0.052258**	**0.038766**	**0.008103**	**8.922895**
Place of Delivery				
Elsewhere*	Base	Base	Base	Base
**Health Facility**	**0.05253**	**0.08735**	**0.01835**	**20.21083**
**Health services immunisation system**				
Money needed to reach health facility				
No	Base	Base	Base	Base
**Yes**	**-0.020596**	**-0.15715**	**0.012947**	**14.255992**
Residual Term			0.054095	60.464386
Total**			**0.09**	**39.535614**

Note: * elsewhere includes respondent’s home, other home, and other places

** residual term consists of the contribution from household wealth status

### Determinants of childhood full vaccination

**Fig. 3:**
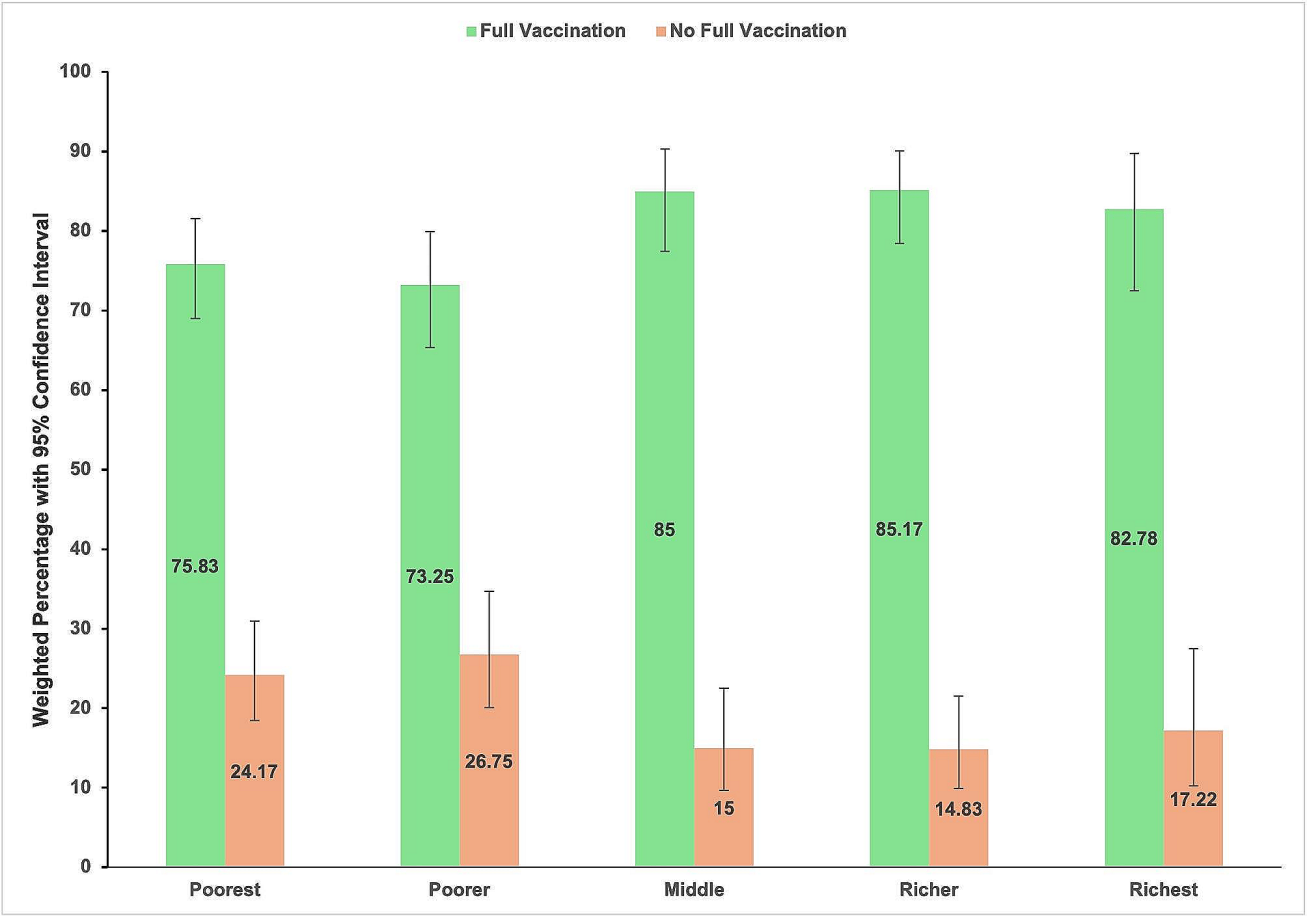
Childhood full vaccination status over the household wealth quintile index in NDHS 2022

Figure 3 depicts the proportion of fully vaccinated children over household wealth index quintiles for the total number of children in the respective quintile. Around three-fourths (75.83%) of the children in the poorest wealth quintile got fully vaccinated compared to 82.3% of the children in the wealthiest counterpart. The graph demonstrates that the proportion of fully vaccinated children increases sharply while moving from the children in the poorest wealth quintile to the middle and richer wealth quintiles, then dropping slightly for children in the richest wealth quintile.

**Fig. 4:**
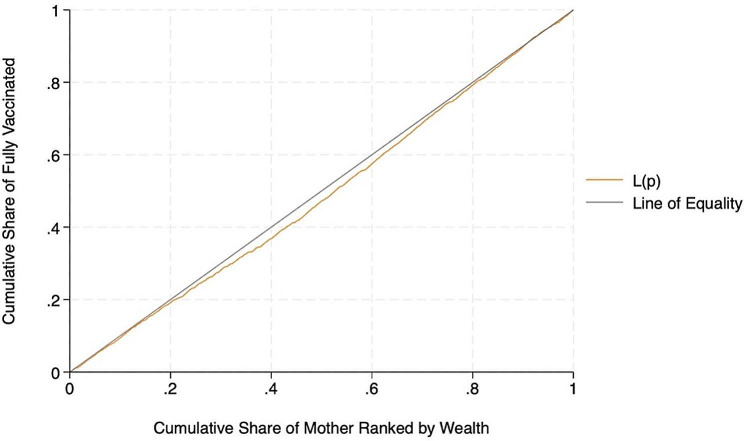
Concentration curve for full vaccination status among children against wealth rank

Figure 4 shows the inequality in fully vaccinated children by wealth status. Since the concentration curve is below the line of equality, the proportion of fully vaccinated children was disproportionately higher among children from wealthy groups. A positive estimated relative CIX of 0.090 (standard error: 0.029; *p* < 0.01) indicates that the proportion of fully vaccinated children was relatively higher among wealthier households than their poor counterparts. However, the overlap of the line of equality and the concentration curve at both ends suggests equality at the extremes. This implies that full vaccination rates are similar among the poorest and wealthiest segments of the population.

### Decomposition of the relative concentration index

The outputs of a decomposition analysis and each determinant’s contribution are depicted in Table 2. The determinants (excluding the wealth quintile) incorporated into the model explained 40% of socioeconomic inequality in childhood full vaccination uptake.

The major contribution to the inequality was from the place of delivery (20.21%), followed by maternal education (16.80%), money to reach health facilities (14.25%), and ANC ≥ 4 visits (8.92%). An explanatory variable such as caste contributed relatively little to the inequality in childhood full vaccination, accounting for only 3.03%. However, the percentage contribution of Dalits within the caste/ethnicity category was the highest (21.28%) for socioeconomic inequality. The residual contribution, including the wealth quintile, was around 60%. Mother’s employment (in the past 12 months) and awareness of HMG meetings in the ward were the factors that caused a reduction in wealth-related inequality by 19.25% and 12.96%, respectively, in the children who were fully vaccinated.

## Discussion

This study analysed the determinants of full vaccination among children in Nepal using data from the NDHS 2022. The odds of Nepalese children being fully vaccinated with respect to variables from the health service immunisation system, communication and information, household characteristics, and paternal attitude, knowledge and practices were measured. Further, wealth-related inequality in fully vaccinated children was computed, and a decomposition analysis was performed to identify the determinants that explain the socioeconomic inequality [43]. Our analysis found that ANC ≥ 4 visits, awareness of HMG meetings in the ward, and household size were major determinants for full vaccination uptake among children. The proportion of fully vaccinated children was disproportionately higher among the children belonging to wealthy households. The concentration index decomposition showed that wealth-related inequality in childhood full vaccination was primarily explained by place of delivery, maternal education, money needed to each health facility, and ANC ≥ 4 visits.

Our study reported the socioeconomic inequality in childhood vaccine uptake from the poorest households. This finding was consistent with previously published studies from Nepal [11, 21]. As observed in earlier analysis conducted using four rounds of the NDHS (2001, 2006, 2011, 2016), the socio-economic inequality concerning full vaccination coverage between the socio-economic groups measured by relative CIX has, on average, narrowed over this period [21]. The relative CIX obtained from these four rounds of NDHS were 0.21, 0.20, 0.08, and 0.054, respectively [21]. The analysis presented in this paper using the data from NDHS 2022 has shown that the relative CIX for full vaccination has slightly increased to 0.090, signalling rise in socioeconomic inequality in vaccination uptake over the last one and half decades. Thus, identifying the factors driving the inequalities is a preliminary step in designing effective policy measures to reduce observed socioeconomic inequality.

The decomposition analysis revealed that maternal health service utilisation inequalities, including antenatal care and institutional delivery, largely explain inequalities in the uptake of full vaccinations. Both factors accounted for about 29% of the total inequality in full vaccination uptake. In Nepal, immunisation services are provided at the same health facility where maternal health care is provided, the utilisation of maternal health care can serve as a proxy indicator for accessibility of health facilities offering immunisation services. Observed inequality could be associated with disparity in access to immunisation services. Although maternal health care services are supposed to be provided free at public health facilities in Nepal, women with low socioeconomic status experience a financial burden for maternity care, as they have to pay not only for transportation, accommodation, and food but also loss earning due to absenteeism from work [57, 58]. This could impede the use of services among socioeconomically disadvantaged populations, especially those who rely predominantly on public health facilities for their health care requirement. Our findings are consistent with the findings from other studies which found antenatal check-ups a significant factor contributing to the inequality in childhood full vaccination [17, 51, 59]. From a policy perspective, targeted efforts are necessary to enhance maternal health service utilisation among the socioeconomically disadvantaged individuals and mitigate the inequality in vaccination uptake. Other studies are also reported a positive association of at least four ANC visits with childhood full vaccination in Nepal [11, 27, 60] and in Bangladesh [61], Indonesia [20], and Nigeria [62].

Furthermore, acute poverty reflected through the inability of the families to visit the health facility was a significant contributor (14.25%) to inequality in childhood full vaccination in this study. This finding corroborates a previous study that analysed inequalities in full childhood vaccination in Nepal based on the NDHS 2016 [60]. This is likely because the Government in Nepal has been offering free vaccines since the initiation of the Expanded Programme on Immunisation in 1979 [8]. Though vaccines are accessible through immunisation clinics throughout Nepal, poverty restricts some women’s ability to reach them. Mothers facing financial challenges were less likely to fully vaccinate to their children. Our findings suggest expanding the demand-side financing program for poor households to reward mothers vaccinating their children, alleviating financial obstacles to vaccination.

The mother’s education accounted for a relatively substantial portion of the inequality in childhood full vaccination (16.79%), which are consistent findings with earlier studies [16, 51]. In addition to improving health literacy, better education for mothers can transform women into financially independent, increase self-confidence, and ultimately empower them to address their and their children’s health needs [63]. Almost one-third (33.67%) of the poorest women had no education, relative to one among eighty women (1.22%) in the highest wealth quintile [64]. That’s why, besides improving women’s education equitably, the NIP can leverage and ensure the involvement of Female Community Health Volunteers (FCHVs) and other community health workers in communicating with problematic households at the community level in collaboration with local government. Previous studies in Nepal also identified maternal education as a potential determinant of full vaccination [19, 26].

It was also observed that caste/ethnicity accounts for some of the socioeconomic inequalities in the uptake of the full vaccines (3.03%) with significant portion of this contribution mainly stemmed from the Dalit caste (21.28%). These children were disproportionately concentrated among mothers from less wealthy households [64]. Meanwhile, the relatively lower literacy rate and sociocultural practice prevalence among the Dalit could be the reason behind the lower rate of full vaccination among the children in that group, as reported in previous studies [11, 32, 60, 65]. Additionally, community engagement can also be ensured through dialogue meetings by mobilizing and influencing persons among targeted groups at the local level to address misconceptions and concerns about vaccinations.

Decomposition analysis of this study also identified the awareness of HMG meetings as an important contributor to the reduction in socioeconomic inequality of full vaccine (-12.95%). The likelihood of full vaccination among children was greater among the children whose mothers were aware of the HMG meeting in their respective wards. This emphasizes the importance of the HMG, community groups led by FCHVs that bring together women of reproductive age (15–49 years) monthly to discuss and promote several areas of health, particularly related to maternal, newborn, and child health [66]. Additionally, we found approximately 60% of the socioeconomic inequality (excluding the wealth quintile) remained unexplained. This was inevitable as we did not consider supply-side factors, which have been explained as potential predictors in other studies for vaccination coverage, such as cold chain maintenance, availability of vaccines, and adequacy of staff in the facility [13, 14]. Furthermore, other demand-side factors might explain the socioeconomic inequality in childhood vaccination uptake, which needs to be assessed in future studies.

### Strengths and limitations of the study

This study has some strengths and limitations. Strengths include rigorous statistical techniques applied to understand childhood full vaccination’s determinants. We applied the decomposition analysis to identify critical determinants of wealth-related inequality measured by the relative concentration index in the country’s childhood full vaccination uptake. However, this study has also some limitations. First, inclusion of the vaccination status of children based on the verbal response of mothers might have introduced differential misclassification into this study due to the potential under-reporting of children who were not fully vaccinated. This is because mothers can provide false reports about the immunization status of their children in the absence of health cards to appear socially acceptable. Second, vaccine stockouts, poor cold chain systems, and the non-readiness of health service providers to administer the vaccines during mothers’ use of maternal health services are some of the health system barriers to the child being fully vaccinated that were not captured in the data used for this study, which could have led to an underestimation of the impact of maternal health service use (ANC and place of delivery) on routine vaccine coverage found in this study. Thirdly, we could not consider the complex survey design while decomposing the socioeconomic inequality in vaccination uptake. This limitation resulted in underestimating the elasticity variance, leading to inaccuracies in the standard error, confidence interval, and *p*-value. However, it does not influence the point estimate since sampling weight was considered during the analysis.

## Conclusion

This analysis found uptake of full vaccination among children is pro-wealthy in Nepal. Policymakers should recognize this disparity and implement equity-oriented policies to support socioeconomically disadvantaged groups. We recommend targeted interventions to enhance maternal healthcare services, financial access to health facilities, and educational attainment among socioeconomically disadvantaged mothers. These interventions can substantially reduce inequality in the childhood full vaccine uptake in Nepal.

### Electronic supplementary material

Below is the link to the electronic supplementary material.

Supplementary Material 1

## Data Availability

Data used in this study are publicly available secondary data obtained from the DHS (https://dhsprogram.com/data/available-datasets.cfm) program.

## Abbreviations

ANC: Antenatal care
AOR: Adjusted odds ratio
BCG: Bacillus Calmette-Guerin
CC: Concentration curve
CI: Confidence interval
CIX: Concentration index
ECIX: Erreygers normalized concentration index
COR: Crude odds ratio
DPT: Diphtheria, Pertussis, Tetanus
EDP: External development partner
FCHV: Female community health volunteers
FIDP: Full immunisation declaration program
GVAP: Global vaccine action plan
HMG: Health mother group
MDG: Millenium development goal
MMR: Measles Mumps Rubella
MR: Measles-Rubella
NDHS: Nepal demographic and health Survey
NIP: National immunisation program
OPV: Oral Polio Vaccine
SDG: Sustainable development goal
VIF: Variance inflation factor
VPD: Vaccine-preventable diseases
WHO: World Health Organisation
