# Supplementary material for: Determinants of wealth-related inequalities in full vaccination coverage among children in Nepal: a decomposition analysis of nationally representative household survey data
Source: BMC Public Health. 2024 Jul 25;24:1990. doi: 10.1186/s12889-024-19456-z (PMC11270921; doi:10.1186/s12889-024-19456-z)
Supplement: Supplementary file 1 — Supplementary Material 1 [file 12889_2024_19456_MOESM1_ESM.docx]

**Supplementary Table 1 : Descriptive Definition of Explanatory Variables**

| **Variables** | **Categories** | **Definition** |
| --- | --- | --- |
| **Household characteristics** |  |  |
| Province | Koshi, Madhesh, Bagmati, Gandaki, Lumbini, Karnali, Sudurpaschim | Seven provinces |
| Place of residence | Urban, Rural | Respondents at the time of the survey live either in rural or urban places. |
| Ecological zone | Hill, Mountain, Terai | Three zones horizontal division |
| Mother’s age | 15-19;20 -24; 25-29;30-34, and 35 and Above | Age of mothers at the time of the interview |
| Caste/Ethnicity | Brahmin, Chhetri, Madhesi, Terai Janajati, Hill Janajati, Dalit, Muslim | Categorized into seven caste/ethnicities, others were merged into the Madhesi caste |
| Maternal Education | No Education, Primary, Some Secondary, & Higher | No education, meaning illiterate, classes (1-8) are in primary, and those from class 9 to 10 are in some secondary, above SLC are in higher categories. |
| Wealth status | Poorest, Poorer, Middle, Richer, Richest | Five quintiles |
| Sex of Child | Male, Female | Sex of the index child |
| Birth order | First, Second, | Birth order of a most recent child |
|  | Third and More |  |
| Household size | 1-3, 4-5, 6+ | Number of members in the sampled household |
| Mother’s employment ( in Past 12 Months) | Yes, No | Mother’s engagement in employment in the past year |
| **Communication and information** |  |  |
| Mother’s exposure to media | Not at All, At Least Once a Week, More Than Once a Week | Frequency of exposure to any mass media (radio/television/newspaper) in a week |
| Awareness of HMG in Ward | Yes, No | Asked respondent about whether they were aware of HMGs in the ward |
| **Parental attitude, knowledge and practices** |  |  |
| ANC ≥ 4 visits | Yes, No | No of respondent’s ANC checkups in last pregnancy |
| Place of Delivery | Health facility, Elsewhere (respondent’s home, other home, others) | Institutional delivery of last birth for respondent(mother) |
| **Health service immunisation system** |  |  |
| Money needed to visit health facility | As Big Problem, Not As a Big Problem | Asked to respondent(mother) about the money needed to visit health facility as a big problem |
| Distance to health Facility | As Big Problem, Not As a Big Problem | Asked to respondent(mother) about accessibility of health facility in terms of distance as big problem |

**Supplementary Table 2: Variance inflation factors of variables used in the study**

| **Variables** | **Variance inflation factors** |
| --- | --- |
| **Household characteristics** |  |
| **Province** |  |
| Madhesh | 2.87 |
| Bagmati | 1.98 |
| Gandaki | 1.61 |
| Lumbini | 1.83 |
| Karnali | 2.63 |
| Sudurpaschim | 2.26 |
| **Ecological Zone** |  |
| Hill | 3.31 |
| Terai | 6.16 |
| **Place of residence** |  |
| Rural | 1.19 |
| **Child Sex** |  |
| Female | 1.01 |
| **Mother's Age(Years)** |  |
| 20-24 | 3.31 |
| 25-29 | 3.66 |
| 30-34 | 3.07 |
| 35-49 | 2.26 |
| **Maternal Education** |  |
| Primary | 2.41 |
| Some Secondary | 2.78 |
| SLC and Higher | 2.71 |
| **Caste/Ethnicity** |  |
| Chhetri Hill | 3.7 |
| Terai Caste | 3.96 |
| Dalit | 3.77 |
| Hill Janajati | 3.56 |
| Terai Janajati | 2.52 |
| Muslim | 2.19 |
| **Wealth status** |  |
| Poorer | 1.68 |
| Middle | 2.05 |
| Richer | 2.21 |
| Richest | 2.31 |
| **Household Size** |  |
| Four-Five | 2.56 |
| Six-Twenty Six | 2.71 |
| **Birth Order** |  |
| Two-Three | 1.62 |
| Four Plus | 2.12 |
| **Mother's employment (Past 12 Months)** |  |
| Yes | 1.32 |
| **Communication and information** |  |
| **Exposure to mass media** |  |
| Less Than Once a Week | 1.71 |
| Atleast Once a Week | 1.93 |
| **Awareness of HMG meeting in ward** |  |
| Yes | 1.27 |
| **Parental attitude, knowledge and practices** |  |
| **Place of delivery** |  |
| Health Facility | **1.23** |
| **ANC >=4 visits** |  |
| Yes | 1.15 |
| **Health service immunisation system** |  |
| **Distance to health facility** |  |
| Big Problem | 1.46 |
| **Money needed to visit HF** |  |
| Big Problem | 1.34 |
| **Mean VIF** | 2.42 |

**Supplementary Table 3: Determinants of full vaccination among children in Nepal, NDHS 2022**

| **Variables** | **COR (95% CI)** | **P -Value** | **AOR (95% CI)** | **P-Value** |
| --- | --- | --- | --- | --- |
| **Household characteristics** |  |  |  |  |
| **Province** |  | <0.001 |  | 0.154 |
| Koshi | 1 |  | 1 |  |
| Madhesh | 0.48 (0.26-0.90) * |  | 0.79(0.32-1.99) |  |
| Bagmati | 1.19(0.52-2.69) |  | 0.89(0.40-1.99) |  |
| Gandaki | 3.34 (1.21-9.23) * |  | 2.37(0.79-7.14) |  |
| Lumbini | 1.37(0.65-2.93) |  | 1.35(0.54-3.37) |  |
| Karnali | 1.27(0.61-2.67) |  | 2.03(0.85-4.84) |  |
| Sudurpaschim | 1.88(0.90-3.95) |  | 1.66(0.63-4.39) |  |
| **Place of Residence** |  | 0.38 |  | 0.408 |
| Urban | 1 |  | 1 |  |
| Rural | 1.06 (0.93-1.21) |  | 0.83(0.54-1.29) |  |
| **Sex of Child** |  | 0.214 |  | 0.379 |
| Male | 1 |  | 1 |  |
| Female | 0.81(0.58-1.13) |  | 0.85(0.59-1.22) |  |
| **Maternal Education** |  | <0.001 |  | 0.905 |
| No Education | 1 |  | 1 |  |
| Primary Education | 2.24(1.33-3.77) ** |  | 1.19(0.67-2.10) |  |
| Some Secondary Education | 3.31(1.89-5.82) *** |  | 1.23(0.64-2.37) |  |
| SLC and Above Education | 3.43(1.76-6.70) *** |  | 1.07(0.49-2.34) |  |
| **Mother’s Age (years)** |  | 0.15 |  | 0.359 |
| 15-19 | 1 |  | 1 |  |
| 20-24 | 1.45(0.76-2.77) |  | 1.07(0.54-2.15) |  |
| 25-29 | 1.55(0.82-2.93) |  | 1.12(0.51-2.48) |  |
| 30-34 | 2.66(1.19-5.93) |  | 2.19(0.75-6.38) |  |
| 35-49 | 1.10(0.44-2.71) |  | 0.86(0.28-2.62) |  |
| **Caste/Ethnicity** |  | <0.001 |  | 0.246 |
| Brahmin Hill | 1 |  | 1 |  |
| Chhetri Hill | 0.63(0.23-1.72) |  | 0.64(0.22-1.81) |  |
| Terai Caste | 0.28(0.10-0.74) * |  | 0.60(0.20-1.82) |  |
| Dalit | 0.23(0.09-0.58) |  | 0.44(0.16-1.20) |  |
| Hill Janajati | 0.67(0.26-1.69) |  | 1.11(0.43-2.89) |  |
| Terai Janajati | 1.00(0.29-3.45) |  | 1.28(0.36-4.56) |  |
| Muslim | 0.20(0.07-0.61) ** |  | 0.54(0.16-1.79) |  |
| **Wealth status** |  | <0.05 |  | 0.160 |
| Poorest | 1 |  | 1 |  |
| Poorer | 0.87(0.53-1.45) |  | 1.00(0.57-1.75) |  |
| Middle | 1.81(1.03-3.17) * |  | 2.08(1.04-4.16) * |  |
| Richer | 1.83(1.03-3.25) * |  | 1.69(0.83-3.41) |  |
| Richest | 1.53(0.77-3.04) |  | 1.16(0.48-2.79) |  |
| **Household size** |  | <0.01 |  | <0.05 |
| Large(six-twenty six) | 1 |  | 1 |  |
| Medium(four-five) | 1.20(0.78-1.85) |  | 0.92(0.57-1.48) |  |
| Small(one-three) | 2.91(1.48-5.72) ** |  | 2.36(1.16-4.78) * |  |
| **Birth Order** |  | <0.001 |  | 0.104 |
| One | 2.53(1.53-4.17) *** |  | 1.41(0.61-3.28) |  |
| Two-Three | 2.44(1.51-3.95) *** |  | 1.95(0.92-4.14) |  |
| Four Plus | 1 |  | 1 |  |
| **Mother’s Employment (Past 12 Months)** |  | <0.01 |  | 0.063 |
| No | 1 |  | 1 |  |
| Yes | 1.80**(1.21-2.66) |  | 1.52(0.98-2.35) |  |
| **Communication and information** |  |  |  |  |
| **Exposure to Mass Media** |  | <0.01 |  | 0.934 |
| No Exposure | 1 |  | 1 |  |
| Less than Once a Week | 1.62(1.02-2.58)* |  | 0.96(0.60-1.53) |  |
| Atleast Once a Week | * |  | 1.05(0.66-1.68) |  |
| **Aware of HMG in ward** |  | <0.001 |  | <0.05 |
| No | 1 |  | 1 |  |
| Yes | 2.08(1.41-3.06) *** |  | 1.71(1.09-2.71) * |  |
| **Parental attitude, knowledge, and practices** |  |  |  |  |
| ANC Visit |  | <0.001 |  | <0.05 |
| Less than Four | 1 |  | 1 |  |
| Four or More | 2.40(1.60-3.60) *** |  | 1.67(1.10-2.53) * |  |
| Place of Delivery |  | <0.001 |  | 0.082 |
| Elsewhere | 1 |  | 1 |  |
| Health Facility | 2.10(1.45-3.04) *** |  | 1.43(0.96-2.14) |  |
| **Health service immunisation system** |  |  |  |  |
| Distance to health facility |  | <0.05 |  | 0.578 |
| Big Problem | 1 |  | 1 |  |
| Not Big Problem | 1.63(1.10-2.41) * |  | 1.14(0.72-1.80) |  |
| Money needed to visit health facility |  | <0.001 |  | <0.05 |
| Big Problem |  |  |  |  |
| Not Big Problem | 1.96(1.36-2.82) *** |  | 1.56(1.02-2.36) * |  |

Note: significant at ***p<0.001, **p<0.01, *p<0.05; COR: Crude Odds Ratio, AOR: Adjusted Odds Ratio

^a^ represents the p-value of the corresponding variable obtained from the single parameter Wald test

^b^ represents the p-value of the corresponding variable obtained from the multiparameter Wald test

**Supplementary Table 4: Decomposition of concentration index (CIX) of childhood full vaccination in Nepal, NDHS 2022**

| **Categories** | **Elasticity** | **Concentration Indices (Determinants)** | **Absolute Contribution to CIX** | **Percentage Contributions** |
| --- | --- | --- | --- | --- |
| **Household characteristics** |  |  |  |  |
| **Maternal education** |  |  |  |  |
| No Education | Base | Base | Base | Base |
| Primary Education | 0.02055237 | -0.0830852 | -0.00683039 | -7.5211529 |
| Some Secondary | 0.02094072 | 0.077222 | 0.00646834 | 7.1224829 |
| SLC and Higher Education | 0.00540828 | 0.41442992 | 0.00896541 | 9.8720856 |
| **Overall** | **0.04690137** | **0.40856672** | **0.00860336** | **9.4734156** |
| **Caste/Ethnicity** |  |  |  |  |
| Brahmin Hill | Base | Base | Base | Base |
| Chhetri Hill | -0.01124485 | -0.08143766 | 0.00366302 | 4.0334584 |
| Terai Caste | -0.0230811 | 0.15944503 | -0.01472066 | -16.209371 |
| Dalit | -0.02600261 | -0.19757254 | 0.0205496 | 22.627795 |
| Hill Janajati | -0.00627062 | -0.11196447 | 0.00280835 | 3.0923549 |
| Terai Janajati | 0.00221053 | 0.13068301 | 0.00115551 | 1.2723723 |
| Muslim | -0.00812669 | 0.18970106 | -0.00616657 | -6.7901962 |
| **Overall** | **-0.07251534** | **0.08885443** | **0.00728925** | **8.0264134** |
| **Mother’s employment (in past 12 months)** |  |  |  |  |
| No | Base | Base | Base | Base |
| **Yes** | **0.03430324** | **-0.12344305** | **-0.01693799** | **-18.650932** |
| **Mother’s age** |  |  |  |  |
| 15-19 | Base | Base | Base | Base |
| 20-24 | 0.00961534 | -0.03494853 | -0.00134417 | -1.480104 |
| 25-29 | 0.01243789 | 0.04599081 | 0.00228811 | 2.5195121 |
| 30-34 | 0.0187875 | 0.1172109 | 0.0088084 | 9.699196 |
| 35-49 | -0.0019732 | 0.00216688 | -0.0000171 | -0.01883232 |
| **Overall** | **0.03886753** | **0.13042006** | **0.00973524** | **10.71977178** |
| **Communication and information** |  |  |  |  |
| **Awareness of HMG meeting in the ward** |  |  |  |  |
| No | Base | Base | Base | Base |
| Yes | **0.02397392** | **-0.14211055** | **-0.01362779** | **-15.005971** |
| **Parental attitude, knowledge, and practices** |  |  |  |  |
| **ANC ≥ 4 visits** | | | | |
| No |  |  |  |  |
| **Yes** | **0.04415168** | **0.03876647** | **0.00684642** | **7.538801** |
| **Place of delivery** | | | | |
| Elsewhere | Base | Base | Base | Base |
| **Health facility** | **0.04592437** | **0.08734622** | **0.01604528** | **17.667948** |
| **Health services immunisation system** |  |  |  |  |
| **Money to reach health facilities** | | | | |
| No | Base | Base | Base | Base |
| **Yes** | **-0.03030601** | **-0.15715024** | **0.01905039** | **20.976961** |
| **Residual Term** |  |  | **0.05299584** | **40.74** |
| **Total** |  |  | **0.0900** | **59.25359222** |
